# Notices and Reviews

**Published:** 1857-10

**Authors:** 


### NOTICES AND REVIEWS.

AMERICAN AGRICULTURIST—New-York, weekly, $1 a year—gives a larger amount of seasonable, reliable agricultural information than any similar publication in the country.

FAMILY GYMNASIUM for the cure of diseases and deformities by certain forms of muscular exercise—by R. T. Trall, M.D., illustrated, by Fowler & Wells, New York—contains a large amount of practically useful information · but this is such a physic-taking age, it is not likely that the sentiments advocated will meet present attention.

THE series of biographical articles of eminent men in LITTELL'S LIVING AGE are of the highest interest to literary men at home and abroad.

GRAHAM'S MAGAZINE—Charles G. Leland, editor—has a steel engraving by Cassewell & Kirnwell, of New York, called *The Jewels*, which any parent might gaze upon by the hour with a pure delight. *Brazil and the Brazilians*, by Rev. Mr. Fletcher, is of high and permanent interest.

ARTHUR' HOME MAGAZINE.—*Old Maid*, by Virginia F. Townsend, and *No.* 3. *Bayard Taylor*, are well worthy of perusal.

GODEY, liberal and courteous always, has not come to hand for months, until now. The frontispiece for October, *The Sisters*, is a lovely picture. It has upwards of fifty illustrations, with a most interesting article on the *Manufacture of Silk—Trials of a Housekeeper, Servant Question. Memories*, by J. W. McJilton, is beautiful.

THE first pages of the *Poughkeepsie Eagle*, and the *American Whig* of Taunton, Mass., are among the best edited of all the secular weeklies we receive. The *Eagle* ought to prize this notice, as we do not remember ever to have seen a "hubbing" of us in its columns.

THE MOTHER'S JOURNAL, $1 a year, New-York, now in its twenty-second vol., has more than one or two truths in the following statement:

"Some of our exchanges we receive with great regularity, and some with great irregularity. *Arthur's Lady's Home Magazine, Lutheran Home Journal, Schoolfellow, Mother's Magazine,* all look well, and are well sustained. *Merry's Museum and Youth's Cabinet* united, forms a most excellent and attractive magazine for the young. *The Inventor* is an admirably conducted scientific paper, of peculiar interest to those who care for the mechanic arts. *The Home, The Lady's Pearl, Mother's Assistant,* reach us sometimes. *The Ladies' Repository* and the *National Magazine* are conducted with marked ability. But nothing pleases us better than HALL'S JOURNAL OF HEALTH. It is *full* of valuable matter.

THE WEST FOURTEENTH STREET COLLEGIATE SCHOOL, now in its thirty-eighth year of successful operation, opens its sessions with high promise, under G. P. Quackenboss, Rector, one of the most thorough instructors in New York; pupils are advanced only as far as their *health will allow*—pity is it that our theological seminaries and other institutions of learning were not conducted on a consideration so politic, so wise, and so humane.

---

### TRAVELLING BY RAIL.

IN repeated journeyings to the West and back, *via* the Pennsylvania Central Railroad, of which J. Edgar Thompson, Esq., is now the efficient President, we have not, in a single instance, failed to make good time, nor have we missed the connection once since its completion. The "night car" is an admirable arrangement, as it allows the wearied traveller to sleep with considerable comfort. We noticed a disposition of the windows in one car, in our last journey, which amounts to a humanity. The arrangement is such, that neither head, arm, nor elbow can be protruded, without a most uncomfortable bodily strain. Another praiseworthy item is, very full time is allowed at Altoona, for breakfast, in going West: and at returning, a breakfast, bountiful, tidy, and well prepared. We commend the Pennsylvania Central, also, for its rigid exclusion of all men from the ladies' night-car, unless they have ladies with them. We write this notice, and of other lines previously, as they merited, never having ridden a mile in a rail-car free of charge.

MARRIED, in Sacramento, Cal., Thursday, May 28, 1857, by Rev. Mr. Baker, Dr. THOMAS J. HALL, of Kentucky, to Miss MARY M., only daughter of R. L. Robertson, Esq.

DIED, on Tuesday, August 18th, 1857, in Kittanning, Pa., at the residence of his son, Rev. J. H. Hall, Principal of the Minnesota Point Seminary, STEPHEN HALL, aged 70 years, a native of Pennsylvania; but from early childhood a resident of Paris, Kentucky, and one of its three oldest citizens. Some years ago, he became a member of the Presbyterian Church, and died in that faith, tearfully praying for pardon, and for "a happy exit into the rest prepared for the people of God." His wife, whom he married forty-eight years ago, survives him, and with his eldest son, the editor of the JOURNAL OF HEALTH, closed his eyes. He was the father, also, of the late S. W. Hall, M. D., of Philadelphia, and of Dr. T. J. Hall, California. Firm in purpose, kindly, and confiding in his nature, he met death with a calm, intelligent courage, retaining a perfect and clear consciousness, almost to the minute of his ceasing to breathe.

